# Use of CO2 Angiography in the Identification of the Bleeding Source of Colonic Diverticular Hemorrhage: A Case Report

**DOI:** 10.1002/jgh3.70276

**Published:** 2025-09-14

**Authors:** Yuya Miyake, Yoshiki Morihisa, Satoko Inoue, Shigeki Arizono, Tetsuro Inokuma

**Affiliations:** ^1^ Department of Gastroenterology Kobe City Medical Center General Hospital Kobe Japan; ^2^ Department of Radiology Kobe City Medical Center General Hospital Kobe Japan

**Keywords:** CO2 angiography, diverticular hemorrhage, iodinated contrast angiography

## Abstract

**Introduction:**

Diverticular hemorrhage is the most common cause of lower gastrointestinal bleeding (LGIB). Because spontaneous hemostasis frequently occurs, identifying the bleeding diverticulum via colonoscopy or iodinated contrast angiography remains challenging. Recently, several reports have demonstrated the utility of CO2 angiography in identifying the bleeding source.

**Case Presentation:**

The patient was a 73‐year‐old male referred to our hospital for hematochezia and was ultimately diagnosed with colonic diverticular hemorrhage. Despite repeated massive hemorrhage, spontaneous hemostasis prevented localization of the bleeding site; neither colonoscopy nor conventional iodinated contrast angiography detected the source. Finally, CO2 angiography was performed to successfully identify the bleeding site, which enabled transcatheter arterial embolization to achieve hemostasis.

**Conclusion:**

In cases of recurrent diverticular bleeding where the bleeding site remains undetectable, CO2 angiography may be an effective method to identify the source and guide targeted therapy.

## 1 Introduction

Diverticular hemorrhage is the most common cause of lower gastrointestinal bleeding (LGIB). In approximately 73%–88% of cases, diverticular bleeding resolves spontaneously, making localization of the bleeding diverticulum via endoscopy challenging [1, 2]. Furthermore, the rebleeding rate is relatively high in cases of colonic diverticular bleeding [2, 3]. Therefore, repeated endoscopic interventions and blood transfusions are often required, resulting in prolonged hospital stays. Even in cases where contrast‐enhanced computed tomography (CT) demonstrated extravasation, the rate of identifying the bleeding diverticulum remains suboptimal [1, 4]. Herein, we report a case of colonic diverticular hemorrhage in which localization of the bleeding site was particularly challenging, resulting in recurrent massive hemorrhages, and was ultimately successfully treated following CO2 angiography‐guided transcatheter arterial embolization.

## 2 Case Report

A 73‐year‐old male with a medical history of hypertension and hyperuricemia, with no previous episodes of LGIB, was referred to our hospital for hematochezia. The patient had no history of smoking or alcohol consumption, and was not taking any regular medications. The patient's vital signs were stable, and the general examination results were normal. Laboratory tests revealed mild anemia (Hb 9.9 g/dL) (Table 1). Contrast‐enhanced CT showed no extravasation of the contrast medium, while multiple diverticula were observed in the colon.

**TABLE 1:** Results of laboratory test.

Hematology		
WBC	51	×102/μL
RBC	301	×104/μL
Hb	9.9	g/dL
Ht	30.7	%
PLT	18.5	×104/μL
Serum chemistry		
TP	5.8	g/dL
ALB	3.4	g/dL
T‐Bil	0.2	mg/dL
AST	18	U/L
ALT	11	U/L
LD	139	U/L
ALP	219	U/L
γ‐GT	8	U/L
AMY	76	U/L
CK	64	U/L
BUN	39.8	mg/dL
CRE	1.49	mg/dL
Na	143	mEq/L
K	4.6	mEq/L
Cl	105	mEq/L
Ca	8.2	mg/dL
CRP	0.01	mg/dL

Colonoscopy was performed after preparation, but bleeding ceased spontaneously, meaning that the bleeding site could not be identified. A few hours after the colonoscopy, the patient experienced massive hematochezia, requiring transfusion of 4 units of packed red blood cells (RBCs), and subsequently underwent repeat colonoscopy.

**FIGURE 1:**
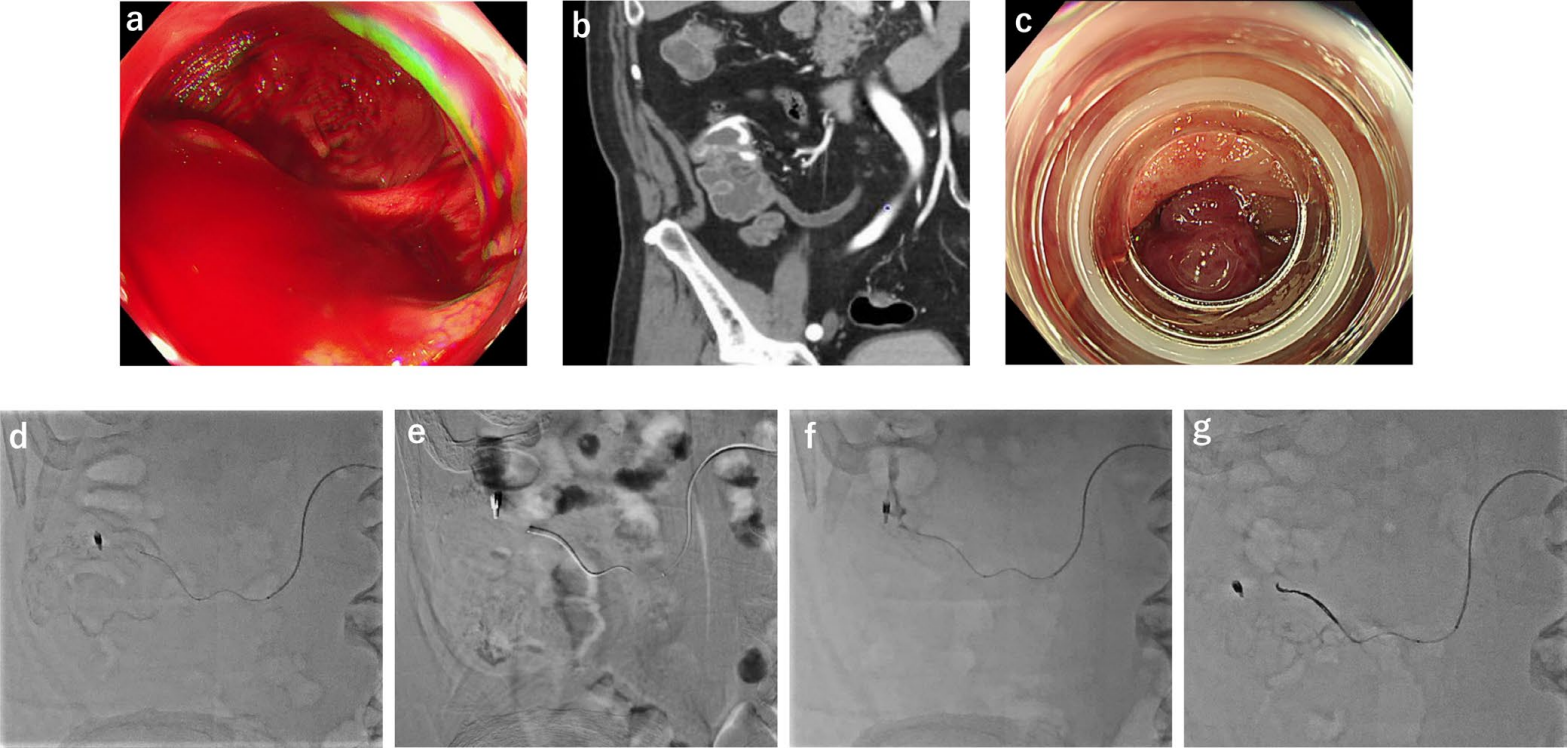
(a) Active bleeding began in the ascending colon, making it difficult to maintain the visual field. (b) Contrast‐enhanced CT revealed extravasation in the ascending colon. (c) Endoscopic band ligation was performed on the diverticulum with an exposed vessel. (d) Initial iodinated angiography revealed no extravasation due to spontaneous hemostasis. (e) CO2 was injected into the artery responsible for bleeding for further assessment. (f) A repeat iodinated contrast study revealed extravasation from the ascending colonic diverticulum. (g) Coil embolization was finally performed on the artery responsible for bleeding.

During colonoscopy, active bleeding started in the ascending colon, making it difficult to maintain the visual field and resulting in hemorrhagic shock (Figure 1a). Therefore, continuation of a safe endoscopic procedure was not possible. After removal of the endoscope, once the patient's vital signs had stabilized, contrast‐enhanced CT revealed extravasation into the ascending colonic diverticulum (Figure 1b). Iodine angiography was subsequently attempted for hemostasis. An approach from the superior mesenteric artery was followed to visualize the artery responsible for the bleeding via the ileocolic branch; however, no extravasation was observed. Ultimately, spontaneous hemostasis occurred, and the procedure was terminated without embolization.

Colonoscopy performed the following day revealed an exposed vessel in the ascending colonic diverticulum. A marking clip was placed near the diverticulum, and endoscopic band ligation was subsequently performed (Figure 1c). However, the next day, the patient experienced another episode of massive hematochezia and hemorrhagic shock, requiring the transfusion of 4 units of packed RBCs. Emergency colonoscopy was subsequently initiated, during which the endoscopic band was found dislodged, and bleeding had already ceased spontaneously. Iodine angiography was performed again using a marking clip as a landmark; however, no extravasation was confirmed (Figure 1d). From the same location, CO2 was manually injected at a flow rate of 2.5 mL/s using a 5 mL syringe (Figure 1e). Following CO2 angiography, iodine angiography was performed again from the same catheter position, immediately revealing extravasation into the colon (Figure 1f). Transarterial coil embolization was subsequently performed at this site (Figure 1g). After coil embolization, no further episodes of rebleeding were observed.

## 3 Discussion

Colonic diverticular hemorrhage is the most common cause of LGIB. Colonoscopy is recommended as the optimal initial diagnostic and therapeutic modality for acute LGIB or colonic diverticular bleeding. However, spontaneous hemostasis rates are relatively high, at approximately 73%–88% [1, 2], while spontaneous hemostasis or disturbed visualization due to blood or fecal material commonly prevents the identification of the bleeding source. Furthermore, the rebleeding rates of colonic diverticular bleeding are relatively high [2, 3], which may result in a prolonged hospital stay and an increased need for blood transfusions. Transcatheter arterial embolization is considered an alternative hemostatic method for patients with continuous bleeding unresponsive to endoscopic treatment.

In general, endovascular treatment for colonic diverticular hemorrhage involves the identification of the bleeding site and embolizing the affected vessel using marking clips placed during endoscopy or extravasation observed on angiography. The success rate of these techniques is 93%–97% when the bleeding source is identified; however, the rate of identifying the bleeding site via angiography can be as low as 24%–48%, particularly when spontaneous hemostasis has already occurred [4, 5, 6, 7].

In this case, the patient experienced repeated episodes of massive bleeding, leading to hemorrhagic shock and difficulties in identifying the bleeding site, despite repeated urgent colonoscopies and iodine angiography. However, successful hemostasis was ultimately achieved using CO2 angiography.

CO2 is generally used as a negative contrast agent for patients with iodine allergies or renal impairment, and has further proven useful in detecting arteriovenous shunts, traumatic bleeding, and gastrointestinal bleeding [8]. The principle lies in the vasodilatory effects of CO2, which increase blood flow and may induce extravasation from the bleeding site [9]. In previous reports, thrombolytics and vasodilators have been used to induce bleeding; however, these approaches carry the risk of bleeding complications [10]. Owing to its localized action and short duration, CO2 injection can reduce the risk of complications.

Furthermore, CO2 is much less viscous than iodinated contrast medium, and can easily fill small and stenotic blood vessels. Consequently, bleeding from small and spastic blood vessels can be visualized [8, 9]. The procedure involved the identification of the diverticulum responsible for bleeding using marking clips, followed by the injection of 5 mL of CO2 from a microcatheter at a rate of 2.5 mL/s, and repeat iodine angiography from the same catheter position [8]. However, if air contamination occurs in this process, there is a potential risk of ischemic complications due to air embolism [9]. This complication is uncommon but clinically significant, and thus should be carefully recognized and monitored.

As demonstrated in this case, when extravasation of the contrast medium is confirmed on contrast‐enhanced CT but endoscopic hemostasis is difficult, CO2 angiography can be an effective method to induce extravasation at the bleeding site to allow identification of the bleeding source. CO_2_ angiography has been suggested to be useful not only for diverticular bleeding but also for other bleeding disorders, including upper gastrointestinal bleeding [9]. However, reports on CO2 angiography in this context are rare, and further accumulation of cases is required to fully determine its efficacy.

## Consent

Informed consent was obtained from the patient for the study and publication of the manuscript.

## Conflicts of Interest

The authors declare no conflicts of interest.

## Data Availability

Data sharing not applicable to this article as no datasets were generated or analyzed during the current study.
